# Traumatic brain injury

**DOI:** 10.1111/cns.13397

**Published:** 2020-05-25

**Authors:** Julian E. Bailes, Cesar V. Borlongan

**Affiliations:** ^1^ Department of Neurosurgery NorthShore University Health System Evanston IL USA; ^2^ Department of Neurosurgery and Brain Repair University of South Florida College of Medicine Tampa FL USA

Traumatic brain injury (TBI) stands as a major cause of mortality and morbidity around the world. More than 30% of injury‐related deaths in the United States involve TBI.^1^, ^2^ TBI entails two phases of injury, namely an initial head trauma via an external force that results in mechanical damage to brain tissue and a secondary biochemical cascades including inflammation, apoptosis, oxidative stress, and other pathophysiological complications that propel further brain degeneration.^3^, ^4^ Treatment options for TBI remain limited, indicating an urgent need to examine novel therapeutic modalities designed to protect or to regenerate the injured brain.

This special issue compiles recent advances in TBI research focused on cell‐based regenerative medicine and pharmacologic therapies. We envision that rigorous translational research will provide the necessary testbed for ushering safe and effective therapeutics from the laboratory to the clinic.

Yasuhara and colleagues^5^ review the field of cell therapy for TBI and a relevant brain disorder, stroke. Here, they identify technical challenges that accompany cell transplantation, such as cell dose, timing, and delivery. In particular, they outline important preclinical investigations that are warranted to translate induced pluripotent stem cells for clinical applications in TBI and stroke.

Next, our laboratory further elucidates the potential benefits, as well as caveats associated with cell therapy for TBI.^6^ This review paper tackles neuroinflammation as a major cell death mechanism that closely approximates the secondary cell death in TBI. We also recognize that because neuroinflammation ensues immediately after TBI onset but also persists over time, it presents a large therapeutic window for cell therapy intervention. We provide encouraging laboratory studies detailing the success of cell transplantation in mitigating the inflammation‐plagued secondary cell death of TBI.

Recognizing that cell therapy may not be a magic bullet, Willing and co‐workers^7^offer a combination therapy. Here, they show the potential of combining cell therapy with drugs that target signaling pathways of the neuro‐immune‐inflammatory axis. The concept posits that since singular targeting of a cell death pathway mostly fails, drugs that engage multiple pathways will likely promote improved sequestration of degenerative processes for TBI.

The next two papers indeed advance novel drugs that encompass multiple targeting of cell death pathways. The paper by Dore and teammates demonstrate the potential of engaging the lipid metabolite prostaglandin E2 (PGE_2_) as therapeutic targets to treat repetitive concussions and other acute brain injuries.^8^ PGE_2_ binding with the EP2 receptor activates adenylate cyclase and phosphorylates various cellular targets can lead to multiple neuroprotective processes, including anti‐inflammation. In a similar fashion, Greig and co‐investigators^9^ reveal that the small molecular weight drug (‐)‐phenserine tartrate (PhenT), originally developed for Alzheimer's disease, effectively abrogates mild and moderate TBI via mitigation of multiple components of programmed neuronal cell death, including oxidative stress, glutamate excitotoxicity, neuroinflammation, and effectively reversed injury‐induced gene pathways leading to chronic neurodegeneration. Both PGE_2_ and PhenT treatments produced motor and cognitive improvements in TBI animals,^8^, ^9^ further increasing their clinical application potential.

Finally, another closely relevant traumatic disease, spinal cord injury (SCI) is included in this issue. The research group of Ibarra reveals that immunization with neural derived peptides possesses neurogenic and regenerative properties which render motor and sensitive recovery in the chronic stage of SCI.^10^ Such combined targeting of neurogenesis and regeneration may be similarly applied to TBI, since the pathology of TBI manifests with impaired stem cell potencies, as recognized in the cell therapy campaign noted in the other papers described here.^5^, ^6^, ^7^

The articles in this special issue probe the pathology of CNS trauma, including TBI and SIC, and begin to understand potential therapeutic modalities. Along this line, the proposed cell therapy^5^, ^6^, ^7^ and drug and immunization treatments^8^, ^9^, ^10^ offer new directions in the management of TBI. These promising therapies warrant serious consideration as we translate them into clinical applications.

## CONFLICT OF INTERESTS

The authors declare no conflict of interest.
